# Successful treatment of pulmonary aspergillosis due to *Aspergillus fumigatus* in a child affected by systemic lupus erythematosus: A case report from Northeastern Iran

**DOI:** 10.1002/ccr3.4248

**Published:** 2021-05-24

**Authors:** Elham Kashefi, Seyed Javad Seyedi, Kamiar Zomorodian, Zahra Zare Shahrabadi, Hossein Zarrinfar

**Affiliations:** ^1^ Department of Parasitology and Mycology School of Medicine Mashhad University of Medical Sciences Mashhad Iran; ^2^ Department of Pediatrics Faculty of Medicine Mashhad University of Medical Sciences Mashhad Iran; ^3^ Basic Sciences in Infectious Diseases Research Center Shiraz University of Medical Sciences Shiraz Iran; ^4^ Allergy Research Center Mashhad University of Medical Sciences Mashhad Iran

**Keywords:** *Aspergillusfumigatus*, child, Iran, systemic lupus erythematosus

## Abstract

A6‐year‐old girl affected to systemic lupus erythematosus with symptoms of fever, weakness, and lethargy, cough, chest pain, and abnormalchest x‐ray. The isolated *Aspergillus fumigatus* was identified using partial calmodulin gene sequencing. Gradual improvement was observed onday 19 of treatment with amphotericinB (50 mg /day).

## Graphical abstract

Amphotericin B (50 mg /day) was effective tocure a6‐year‐old girl with systemic lupus erythematosus affected to a pulmonary infection with *Aspergillus fumigatus*.

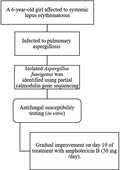

## 1 INTRODUCTION

A 6‐year‐old girl affected with systemic lupus erythematosus with symptoms of fever, weakness, and lethargy, cough, chest pain, and abnormal chest X‐ray. The isolated *Aspergillus fumigatus* was identified using partial calmodulin gene sequencing. Gradual improvement was observed on day 19 of treatment with amphotericin B (50 mg/day).

Systemic lupus erythematosus (SLE) is a chronic autoimmune disorder that can be severe and life‐threatening. It is an inflammatory disease with unknown cause that manifests as kidney, skin‐mucosal, joint, blood, cardiopulmonary, neurological, or immunological involvement.^1^ Death in SLE patients may be due to organ involvement, treatment complications, or cardiovascular disorders.^1^ Infections are one of the leading causes of death in children and adults with SLE.^2^, ^3^ Pulmonary aspergillosis (PA) is a fungal infection caused by *Aspergillus* species, usually associated with primary immunodeficiency, transplant patients, and the use of immunosuppressive drugs.^4^, ^5^, ^6^ However, PA is rarely reported among juvenile systemic lupus erythematosus (JSLE) patients.^7^, ^8^ More than 90% of patients with PA have a history of corticosteroid treatment, immunosuppressive and broad‐spectrum antibiotics therapies, and acute granulocytopenia.^3^ According to some studies, the mortality rate of untreated PA is approximately 100% and the life expectancy in patients treated with amphotericin B is approximately 34%.^9^ Effective management of PA requires rapid and accurate diagnosis based on clinical signs and paraclinical findings. In some cases, high‐resolution computed tomography (CT) scan of the lungs can be a diagnostic method for PA among hematologic patients.^10^ We describe a case of *A fumigatus* isolated from a PA in a child affected by SLE that was successfully treated with a systemic amphotericin B.

## 2 CASE REPORT

**FIGURE 1:**
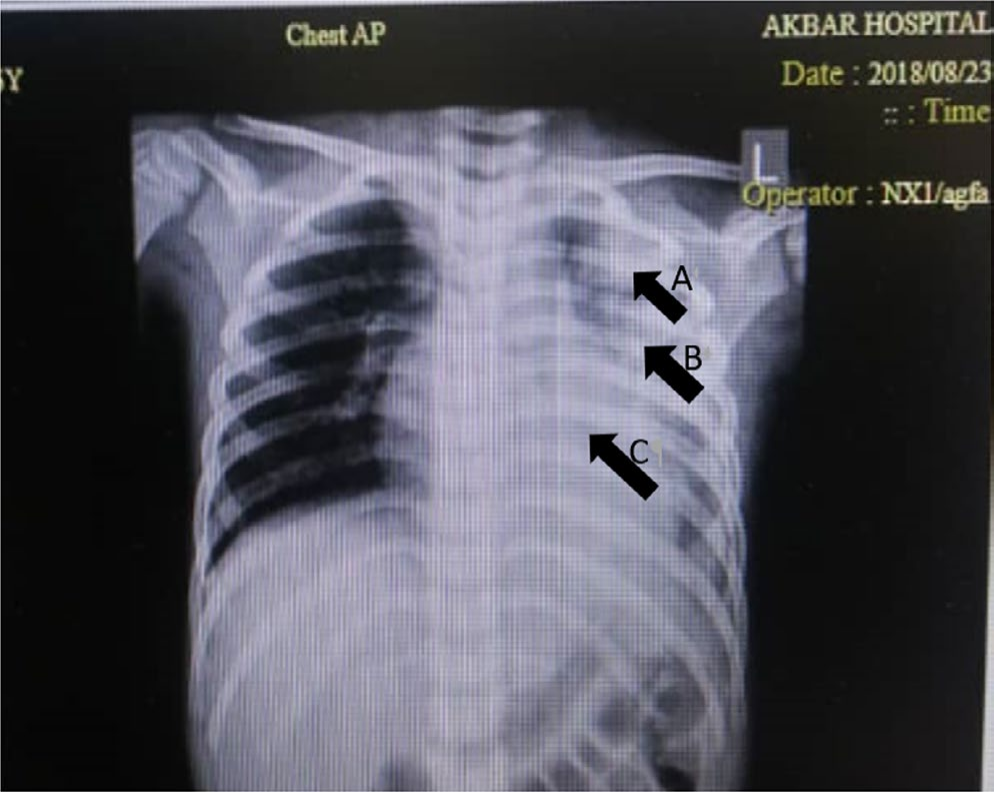
Chest X‐ray revealed consolidation in the left lung, arrow A and B, and it manifested collapse, in the left lung, arrow C

**FIGURE 2:**
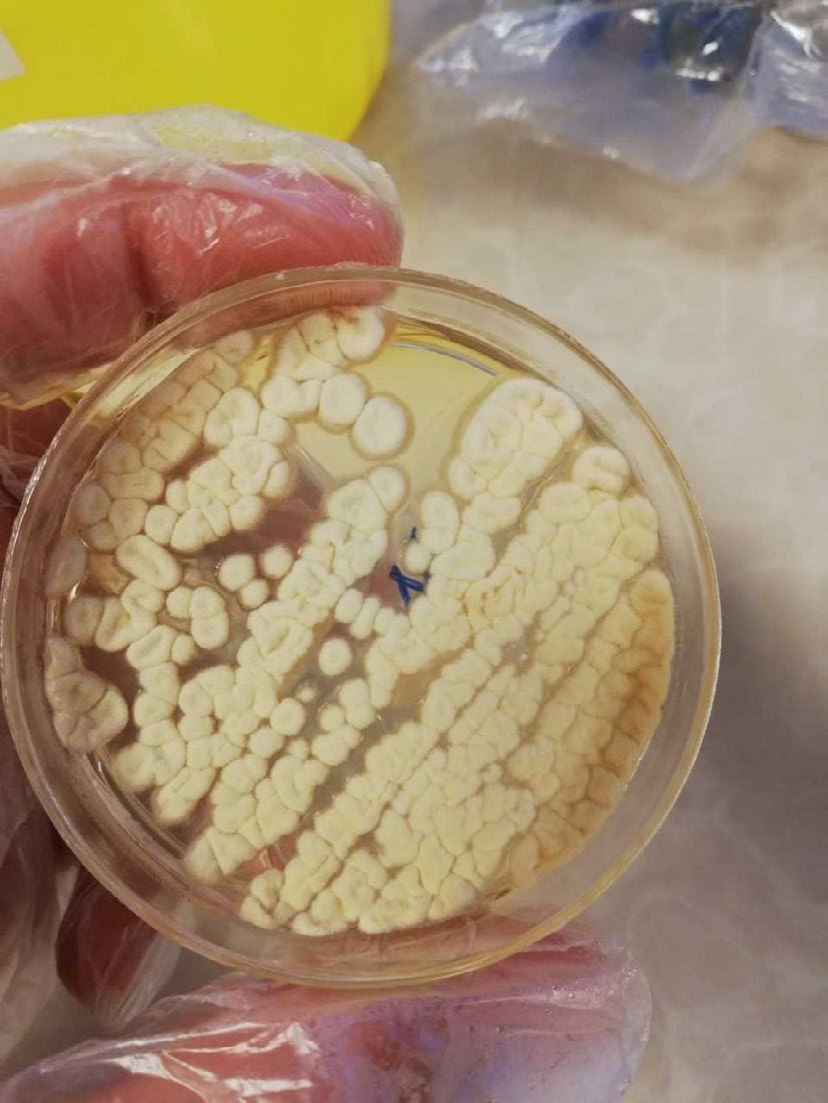
Appearance of *Aspergillus fumigatus* colonies on sabouraud dextrose agar with chloramphenicol (SC)

A six‐year‐old girl living in Mashhad, Iran, has been suffering from SLE since she was 5 years old. In August 2018, she was admitted to Akbar Pediatric Hospital in Mashhad due to a week‐long fever, and then generalized body pain, weakness and lethargy, accompanied by cough and chest pain. Moreover, she was suffering from abdominal pain, complaining of severe and intermittent headache in the frontal or retro‐orbital area along with nausea, vomiting and diarrhea. The chest radiograph appearance showed effusion pleural effusion, pneumothorax, consolidation with dimensions of 43 × 66 mm, and collapse in the left lung (Figure 1). Also, abdominal ultrasound showed hepatomegaly and bowel dilatation in large intestine. Her laboratory and hematological findings showed white blood cell (WBC) = 12.8 × 10⁹/L, red blood cell (RBC) = 3.8 × 10^12^/L, hemoglobin (Hb) = 10.7 g/dl, hematocrit (Ht) = 33.1%, platelet (Plt) = 460 × 10^9^/L, neutrophil (Neu)=51%, lymphocyte (Lym) = 40%, sweat test Na=56 mEq/L, sweat test Cl=38 mEq/L, pH=7.3, PCO2=47 mm Hg, PO2=34.8 mm Hg, HCO3=22.5 mEq/L, blood culture=after 48 hours was negative, complement level C3 = 49 mg/dL, C4 = 12 mg/dL. The patient took steroid medications such as budesonide, hydrocortisone, prednisolone (5 mg/day for 2 days), and also hydroxychloroquine (100 mg / day for 2 days), and amikacin. One week after admission and taking the mentioned drugs, the patient's laboratory results were as follows: WBC=11.1 × 10⁹/L, RBC=4.6 × 10^12^/L, Hb=12.8 g/dL, Ht=38.7%, Plt=390 × 10⁹/L, Neu=64%, Lym=29%, urea=42 mg/dL, creatinine=0.8 mg/dL). One month after admission and taking steroid drugs, due to increased respiratory symptoms, C‐reactive protein (CRP) = 67 mg/dL, and erythrocyte sedimentation rate (ESR) = 34 mm/h, the physicians suspected an infection. The bronchial mucosa was examined using a fiber‐optic bronchoscopy that revealed erythematous, inflamed, thick, and abundant discharge. Moreover, degenerated mycelium was observed in direct microscopic examination with 15% potassium hydroxide (KOH). Direct smear of bronchoalveolar lavage (BAL) specimen did not show fast acid bacilli, and culture was negative for *Mycobacterium tuberculosis* (BK) and other bacteria. The BAL fluid was cultured on sabouraud dextrose agar with chloramphenicol (SC) (Merck, Germany) medium at 35°C for 5 days. The color of the grown colonies was white and wrinkled initially and then turned to green‐turquoise (Figure 2). Microscopic characteristics demonstrated smooth‐walled conidiophores, and terminate in a clavate vesicle in the absence of metulae, uniseriate and the rounded conidia which was classified as *Aspergillus* species. Therefore, this disease was categorized according to EORTC guidelines as probable IPA. After DNA extraction (DENAzist Asia, Mashhad, Iran) according to the manufacturer's instructions, the final identification was performed using partial calmodulin (CaM) gene sequencing using two primer pairs, namely cmd5 (CCGAGTACAAGGAGGCCTTC) and cmd6 (CCGATAGAGGTCATAACGTGG).^11^ The genome sequence of the isolated *Aspergillus* was identified using BLAST algorithm (NCBI) and submitted to the GenBank under the accession number MW459247 as *A fumigatus*. Antifungal susceptibility testing was performed according to the CLSI M38‐A2 broth microdilution method guidelines, using four antifungals, namely itraconazole, voriconazole, fluconazole, and caspofungin.^12^ The antifungal minimum inhibitory concentration (MIC) values of itraconazole, voriconazole, fluconazole, and caspofungin were estimated 1, 0.25, 64, and 4 µg/mL, respectively.

**FIGURE 3:**
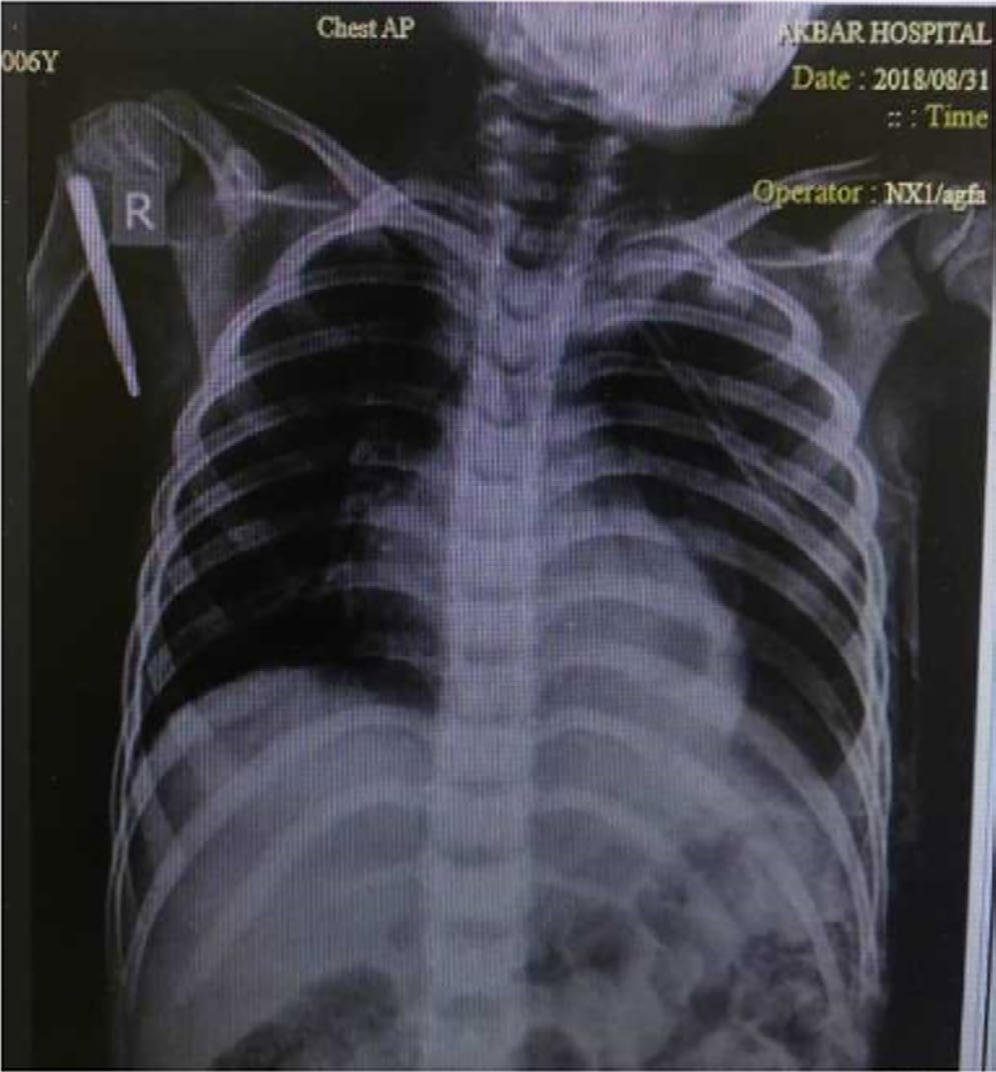
Chest X‐ray revealed gradual recovery in the lung left, after chest tube and treatment with amphotericin B

Following, amphotericin B injection (50 mg/day) was prescribed for 19 days to treat this patient. With the start of taking the amphotericin B, the patient's gradual recovery continued, which was even seen on the chest radiograph appearance (Figure 3). Finally, the patient was discharged after 41 days of hospitalization with a relative improvement.

## 3 DISCUSSION

PA is one of the opportunistic fungal infections in susceptible patients with immune disorders that has increased in recent years.^4^, ^5^, ^6^, ^13^ Among patients with autoimmune diseases, individuals with SLE have predisposing factors for the spread of infection, which leads to death in the first year after diagnosis, approximately with a mortality of 20%‐55%.^1^, ^14^ Predisposing factors for infection in SLE patients include immunodeficiency, low complement levels, lymphopenia, neutropenia, and immunosuppressive drugs include steroid drugs.^14^ In the study, the serum levels of complement C3 and C4 were 49 and 12 mg/dL, respectively, which was lower than the normal level of complement. This finding is similar to a 2012 study by Camila Franca *et al* in Brazil on C3 depletion. Their patient was a 14‐year‐old girl with JSLE with a C3 level of 13 mg/dL.^15^ Although most infections in SLE are caused by bacterial pathogens, new articles demonstrate an increasing incidence of invasive fungal lung infections.^9^, ^16^ In addition, SLE patients with fungal infections have a poorer prognosis than other individuals without SLE.^9^ The pattern of prescribing antifungal drugs in children with aspergillosis is different from that in adults, so more care should be taken in their diagnosis and treatment.^17^ According to some results, mortality in untreated PA in SLE patients is approximately 100% and the life expectancy among patients treated with amphotericin B is approximately 34%.^9^ Amphotericin B alone or in combination with 5‐fluorocytosine or rifampin is the mainstay of treatment for PA, but the duration of treatment is still unknown.^18^ In our patient, after 19 days of administration and use of amphotericin B, a gradual improvement in clinical condition and radiography was observed. Although the liposomal form of amphotericin B has fewer side effects, it could not be used in this governmental hospital due to its high price and lack of availability. In this patient, after taking immunosuppressive drugs such as hydrocortisone, prednisolone, budesonide palmicort, the count peripheral blood lymphocytes decreased to 11% and leukocytes to 1.7 × 10⁹/L. In the VBG test on the first day of hospitalization, the high level of PCO2 (47 mm Hg) was probably due to incomplete ventilation and respiratory distress in the patient. As a result, the concentration of CO2 in the blood increased and the patient developed respiratory acidosis, which following medical procedures the PCO2 decreased to 42.3 mm Hg. Indeed, CRP has been traditionally utilized as a marker of infection and often in excess of 50 mg/dL can indicate the presence of infections in SLE patients. The level of this marker in the patient was 67 mg/dL, which was suspected to have an infection due to respiratory symptoms and radiographic images. Although *C albicans* and *P jiroveci* are common fungal agents in patients with SLE, *Aspergillus* species are also rarely reported.^3^, ^7^, ^19^ In a study by Silva *et al* in Brazil (2012) on patients with JSLE, 2.1% had acute PA.^8^ More than 90% of patients with invasive PA have a history of corticosteroid use, immunosuppressive drugs, acute granulocytopenia, or broad‐spectrum antibiotics.^3^, ^19^ However, mortality in PA depends on the degree and duration of granulocytopenia, the organs affected, and the treatment strategies. Bronchoscopy is one of the effective diagnostic methods in these patients, which was also used in this study. High‐resolution CT scan of the lungs can be a helpful diagnostic method for PA, which in this case showed an effusion pleural effusion along with pneumothorax, and consolidation due to the infection. However, one of the limitations that can be mentioned in this study is the unavailability of lung tissue biopsy for pathological examinations. In this study, calmodulin gene was used to identify the *Aspergillus* isolate, which is one of the effective genes in the accurate identification of different species in this genus. An intriguing point in our study was that the isolated *A* *fumigatus* was resistant to caspofungin based on results of in vitro assays, which show the importance of antifungal resistance among isolates of *A fumigatus* in Iran.^20^, ^21^ Thus, this issue can be a concern, and therefore, experimental treatments should be limited except in special cases.

## 4 CONCLUSION

A correct diagnosis of PA and timely treatment can relieve their symptoms and decrease complications. Thus, correct differentiation between opportunistic fungal agents should be considered, and this case can be an interesting topic to start a more comprehensive study in this group of patients.

## CONFLICT OF INTEREST

The authors declare that they have no conflicts of interest.

## AUTHOR CONTRIBUTIONS

EK: contributed to this manuscript by specimen collection and routine laboratory examinations and writing the manuscript. SJS: contributed to the patient's care; initially diagnosed the patient; and performed the follow‐up care. KZ and ZZS: contributed to this manuscript by identifying fungi, studying the antifungal susceptibility. HZ: (corresponding author) contributed to this manuscript by collecting information for the case report. All authors: reviewed and approved the final manuscript.

## ETHICAL STATEMENT

This case was approved with an Ethics Committee code: IR.MUMS.MEDICAL.REC.1397.576.

## Data Availability

Written informed consent was obtained from patient and was available for provision to the journal on demand.
